# Agenesis of the Left Hepatic Lobe Accompanied by Esophagogastric Cancer: A Case Report and Literature Review

**DOI:** 10.7759/cureus.23960

**Published:** 2022-04-08

**Authors:** Yutaro Yoshimoto, Hajime Orita, Suguru Yamauchi, Sanae Kaji, Tetsu Fukunaga

**Affiliations:** 1 Department of Upper Gastrointestinal Surgery, Juntendo University Hospital, Tokyo, JPN

**Keywords:** liver, esophagogastric cancer, robotic surgery, left hepatic lobe, agenesis

## Abstract

Agenesis of the left hepatic lobe is an exceedingly rare morphological anomaly. Moreover, agenesis of the left hepatic lobe accompanied by esophagogastric cancer is even rarer, with no reports to date. Agenesis of the hepatic lobe is commonly related to some anatomical variations of the gastrohepatic system. A 76-year-old man was referred to our hospital for surgery for esophagogastric cancer with short Barrett’s esophagus. Multiple preoperative imaging modalities revealed agenesis of the left hepatic lobe accompanied by esophagogastric cancer. Robotic proximal gastrectomy and transhiatal lower esophagectomy were performed. Intraoperative findings showed agenesis of the left hepatic lobe. The patient’s postoperative course was favorable. Today, 16 months after surgery, the patient is alive without recurrence of esophagogastric cancer. We report a case of agenesis of the left hepatic lobe in a patient undergoing robotic proximal gastrectomy and transhiatal lower esophagectomy for esophagogastric cancer. Preoperative comprehension of various visceral anomalies reduces the risk of surgical complications.

## Introduction

Agenesis or hypogenesis of the left hepatic lobe is a rare morphological anomaly [1]. It is defined as the absence of liver tissue on the left side of the gall bladder, bordered by falciform ligament [2]. Since agenesis of the hepatic lobe is asymptomatic, it is generally noted incidentally on imaging or autopsy [3,4]. It is commonly associated with anatomical variations of the gastrointestinal and hepatobiliary systems, such as displacement of the gallbladder or volvulus of the gastrointestinal tract [5]. Robotic surgery is advantageous for a transhiatal approach with stable, high-definition views of tight lower mediastinal space. Precise anatomy should be obtained preoperatively by multiple imaging modalities for safe robotic surgery. Herein, we report a successful robotic proximal gastrectomy and transhiatal lower esophagectomy in a patient with agenesis of the left hepatic lobe accompanied by esophagogastric cancer (EGC).

## Case presentation

**Figure 1:**
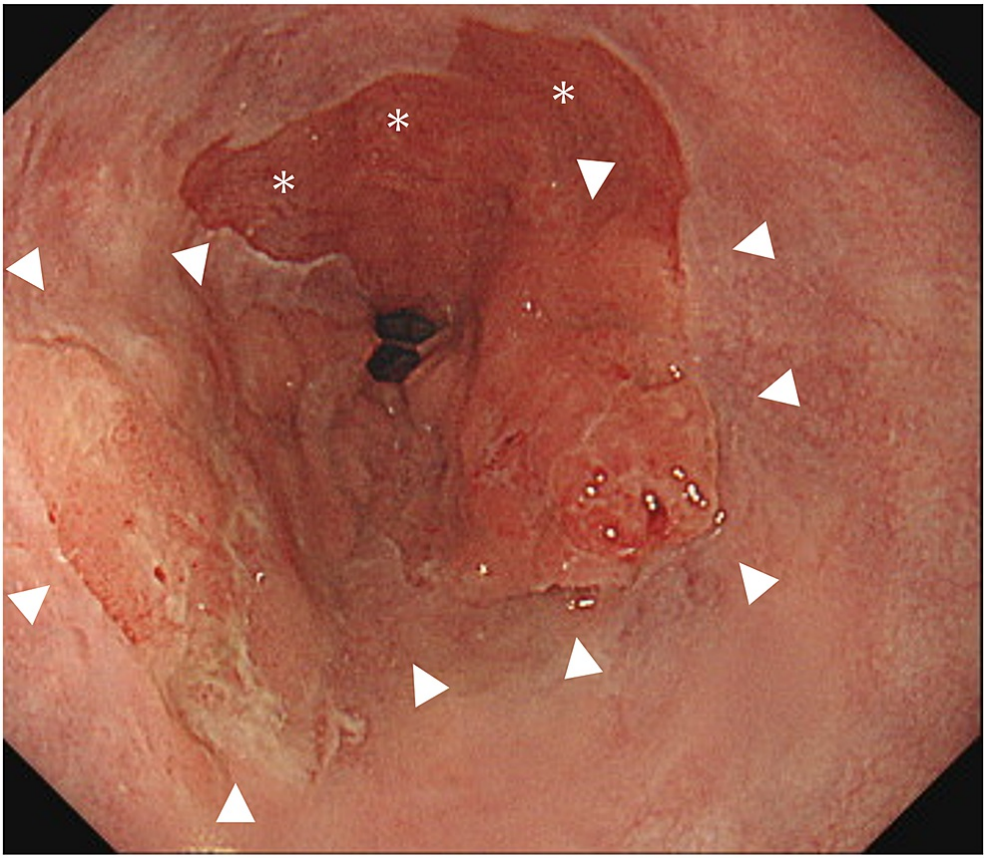
Esophagogastroduodenoscopy disclosing Type 0-Ⅱa-shaped tumor (white arrowheads) at the esophagogastric junction with short-segment Barrett’s esophagus (white asterisk mark).

A 76-year-old man presented to the clinic with epigastralgia of two weeks duration. Esophagogastroduodenoscopy revealed a type 0-Ⅱa-shaped tumor (30 mm in diameter) at the esophagogastric junction with short-segment Barrett’s esophagus (Figure 1).

**Figure 2:**
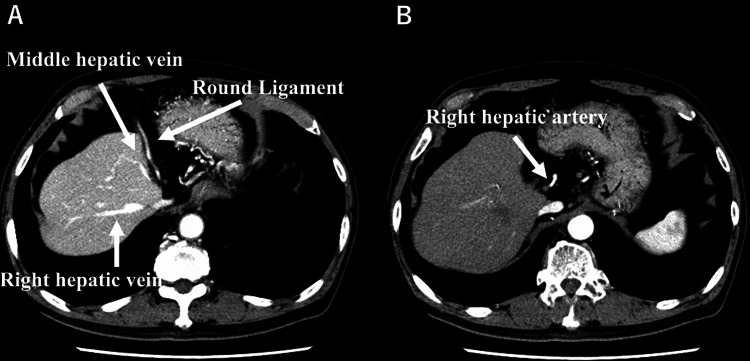
Abdominal CT scan with contrast showing (A) absence of the left hepatic lobe in the late phase and (B) absence of the left hepatic artery in the arterial phase.

Histological findings demonstrated a well-differentiated adenocarcinoma in the mass. The patient was referred to our department for surgery. His medical history included angina pectoris and diabetes mellitus, which were well-controlled. Hematological and biochemical findings were all within normal limits. The Child-Pugh classification was A. Serum levels of tumor markers (carcinoembryonic antigen [CEA], CA19-9, and CA-125) were also within normal limits. Contrast-enhanced abdominal computed tomography (CT) revealed the absence of the left hepatic lobe without obvious esophagogastric tumor or adjoining lymphadenopathy. In the arterial phase of CT, the left hepatic artery and portal vein were not visualized (Figures 2A, 2B). 

**Figure 3:**
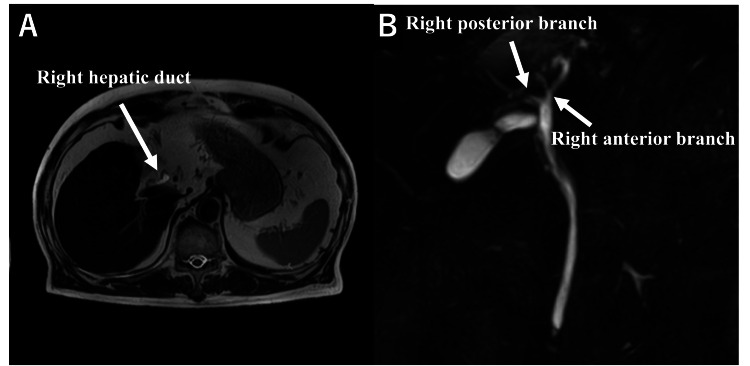
Magnetic resonance cholangiopancreatography (MRCP) detecting (A) agenesis of the left hepatic lobe with (B) complete absence of the left biliary system.

Magnetic resonance cholangiopancreatography (MRCP) also showed agenesis of the left hepatic lobe, with a complete absence of the left biliary system (Figures 3A, 3B).

**Figure 4:**
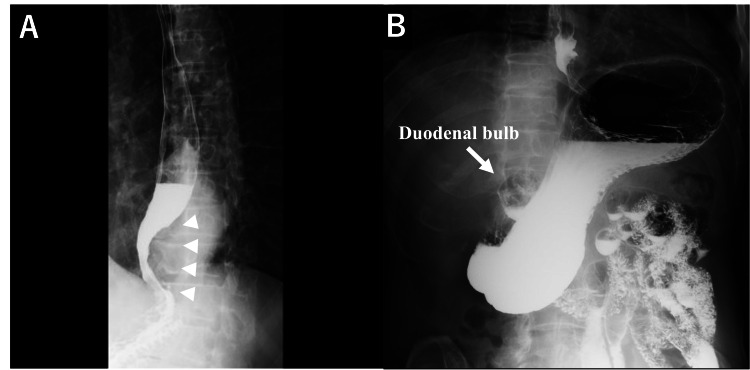
Upper gastrointestinal series representing (A) barium translucency at the esophagogastric junction. (B) Gastric volvulus and high positioning of the duodenal bulb were ruled out.

The upper gastrointestinal series showed barium translucency at the esophagogastric junction. Gastric volvulus and high positioning of the duodenal bulb were ruled out (Figures 4A, 4B).

**Figure 5:**
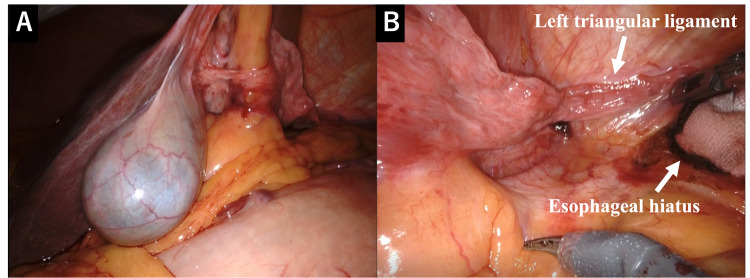
Intraoperative findings revealing (A) agenesis of the left hepatic lobe with no cirrhosis of the right hepatic lobe. (B) The caudate lobe was absent.

The preoperative diagnosis was Siewert II EGC accompanied by agenesis of the left hepatic lobe. The clinical stage of EGC was T2N0M0, stage I. Robotic proximal gastrectomy and transhiatal lower esophagectomy were also performed. Intraoperative findings revealed agenesis of the left hepatic lobe, but the caudate lobe was also not detected. The right hepatic lobe showed no evidence of cirrhosis. Since there was no need to retract the liver during surgery, a transhiatal procedure for the lower mediastinum was performed with good visibility (Figures 5A, 5B), followed by double-tract reconstruction which consists of three stapled anastomoses: esophagojejunostomy, gastrojejunostomy and jejunojejunostomy.

**Figure 6:**
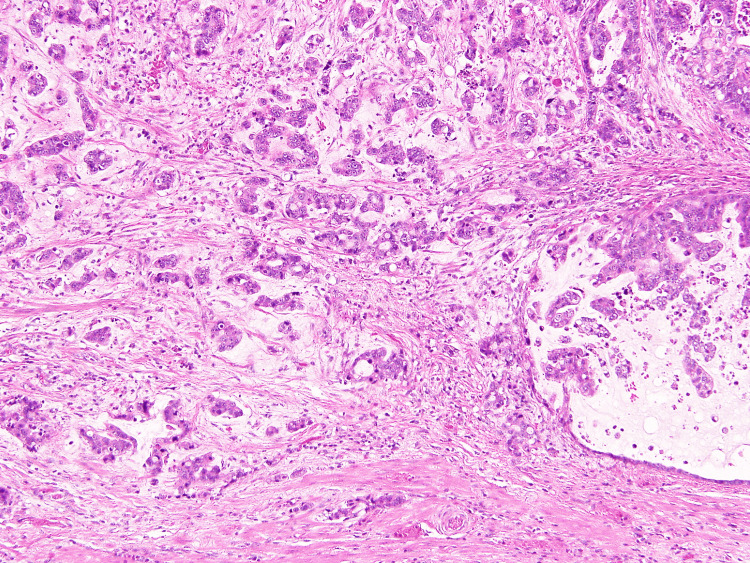
Pathological findings of the tumor showed a well-differentiated adenocarcinoma (hematoxylin and eosin 200x).

Pathological findings of the tumor showed a well-differentiated adenocarcinoma with short-segment Barrett’s esophagus (Figure 6).

The operative time was 613 minutes and the estimated blood loss was 130 mL. 17 lymph nodes were retrieved including one metastatic lymph node at the posterior mediastinal region. The patient’s postoperative course was uneventful. The patient was discharged on a postoperative day 28 without post-surgical complications or gastric reflux symptoms. Today, 16 months after surgery, the patient is alive without recurrence of EGC.

## Discussion

Hepatic lobe agenesis is a rare liver anomaly. It is defined as the absence of liver tissue to the left of the gallbladder fossa [1,2]. In a previous study, agenesis of the left hepatic lobe has a reported incidence of 0.005% among 19,000 autopsy cases [4]. This finding was initially reported in 1932 by Arnold and Ashley-Montagu [6].

Agenesis of the liver is clinically asymptomatic, without laboratory signs of liver dysfunction. Multiple imaging modalities play a significant role in the diagnosis [7]. Contrast-enhanced CT or MRCP enables visualization of the entire liver anatomy, including biliary and vascular anomalies. In this case, abdominal CT for the staging of malignancy incidentally detected the left hepatic lobe defect. Combined with MRI, no left hepatic vessels, portal vein, or biliary system were observed. The caudate lobe was absent, and the right hepatic lobe was not hypertrophic or cirrhotic.

Agenesis of the left hepatic lobe is commonly linked to visceral abnormalities, such as displacement of the gallbladder, high positioning of the duodenal bulb, sigmoid volvulus, and Chilaiditi syndrome, due to deficiency of gastrointestinal fixation and abnormalities of the biliary system [4,8]. It is crucial for surgical planning to confirm such anatomical disorders to ensure a safe operation. In this case, an upper gastrointestinal series was performed to rule out the high positioning of the duodenal bulb, which was absent.

The etiology of agenesis of the hepatic lobe involves congenital factors (an aberrant umbilical vein and abnormal portal development with thrombosis during the prenatal period). And acquired factors (liver cirrhosis, tumor-related vascular occlusion, and Budd-Chiari syndrome) should be ruled out [9-11]. In this case, because there was no cirrhosis or hypertrophy of the right hepatic lobe, a diagnosis of congenital agenesis of the left hepatic lobe was made.

With reference to previous reports, 35 cases of agenesis of the left hepatic lobe have been reported. There are 24 males and 11 females aged 11-79 years at the time of diagnosis. The median age at diagnosis was 59.5 years [11-14]. Surgery was required in 23 cases (66%), including 19 laparotomy cases and four laparoscopic surgery cases. Robotic surgery was performed for the first time in this case. Malignant comorbidities were confirmed in 11 patients (31%), including gastric cancer (four patients), hepatocellular cancer (three patients), pancreatic cancer (one patient), colon cancer (one patient), and cholangiocarcinoma (one patient). This was the first case of agenesis of the left hepatic lobe accompanied by EGC. Although the relevance between agenesis of the hepatic lobe and malignancy is still unknown, it is important to search for malignancy in cases of agenesis of the left hepatic lobe [11].

Proximal gastrectomy and transhiatal lower esophagectomy are standard surgeries for Siewert II-type EGC [15]. In general, retraction or mobilization of the left hepatic lobe is necessary in transhiatal lower esophagectomy. In this case, because of agenesis of the left hepatic lobe, lower esophagectomy and lymphadenectomy of the lower mediastinum were performed with good visibility. Moreover, robotic surgery allowed delicate motion in tight lower mediastinal area using multidimensional stereoscopic visualization and excellent instrument dexterity with tremor filtration, which is advantageous for transhiatal approach compared to laparoscopic surgery.

## Conclusions

This is the first case of agenesis of the left hepatic lobe accompanied by EGC in a patient undergoing robotic surgery. Multiple imaging modalities enabled confirmation of the anatomy associated with hepatic lobe agenesis. Robotic gastrectomy and transhiatal procedures were performed in a good surgical field. For safe surgical planning, it is beneficial to recognize precise anatomical variations preoperatively.

## References

[1] Sato N, Kawakami K, Matsumoto S (1998). Agenesis of the right lobe of the liver: report of a case. Surg Today.

[2] Omeci T, Erdogan ST, Omeci A, Aygun C (2016). A rare congenital liver anomaly: hypoplasia of left lobe. Jpak Med Assoc.

[3] Nacif LS, Buscariolli Ydos S, D'Albuquerque LA, Andraus W (2012). Agenesis of the right hepatic lobe. Case Rep Med.

[4] Merill GG (1946). Complete absence of the left lobe of the liver. Arch Pathol (Chic).

[5] Belton RL, VanZandt TF (1983). Congenital absence of the left lobe of the liver. Radiology.

[6] Matusz P (2010). Extra- and intra-hepatic vascular anatomy in the agenesis of the left lobe of the liver. Clin Anat.

[7] Prithishkumar IJ, Kanakasabapathy I (2010). Agenesis of the left lobe of liver - A rare anomaly with associated hepatic arterial variations. Clin Anat.

[8] Abe H, Yamao M, Yukawa T (2005). A case of agenesis of the left hepatic lobe complicated with hypogenesis of the anterior segment of the right hepatic lobe (Article in Japanese). Nihon Shokakibyo Gakkai Zasshi.

[9] Suzuki T, Sakurabayashi S, Yoshino K (1996). A case of the hypoplasia of the hepatic left lobe complicated with cholangiocarcinoma (Article in Japanese). Nihon Shokakibyo Gakkai Zasshi.

[10] Benz EJ, Baggenstoss AH (1952). Atrophy of the liver. Arch Pathol.

[11] Narita H, Kubota H, Uematu T (2001). A case of hypoplasia of the left hepatic lobe. J Jpn Surg.

[12] Minamoto K, Yuasa I, Ikeda T (2014). Agenesis of the left hepatic lobe undergoing hepatectomy for hepatocellular carcinoma. Jpn J Gastroenterol Surg.

[13] Matsushita K, Gotoh K, Eguchi H (2017). Agenesis of the left hepatic lobe undergoing laparoscopic hepatectomy for hepatocellular carcinoma: a case report. Surg Case Rep.

[14] Kanwal R 3rd, Akhtar S (2021). Left hepatic lobe agenesis with ectopic gallbladder. Cureus.

[15] Kurokawa Y, Takeuchi H, Doki Y (2021). Mapping of lymph node metastasis from esophagogastric junction tumors: a prospective nationwide multicenter study. Ann Surg.

